# Thymus CT-grading and rebound hyperplasia during COVID-19 infection: a CT volumetric study with multivariate linear regression analysis

**DOI:** 10.1186/s43055-022-00784-2

**Published:** 2022-05-16

**Authors:** Ahmed Samir, Rim Aly Bastawi, Ayman Ibrahim Baess, Rania Ahmed Sweed, Omnia Ezz Eldin

**Affiliations:** 1 Department of Radio-Diagnosis, Faculty of Medicine, University of Alexandria, Alexandria, Egypt; 2 Department of Chest Diseases, Faculty of Medicine, Alexandria University, Alexandria, Egypt; 3 Department of Radio-Diagnosis, Faculty of Medicine, University of Alexandria, Alexandria, Egypt

**Keywords:** COVID-19, MDCT, Thymic rebound, CT-severity score

## Abstract

**Background:**

The importance of thymic CT-grading and presence of thymic rebound hyperplasia during COVID-19 infection were only investigated in a few studies. This multivariate study aims to evaluate the relation between thymus CT-grading and rebound during COVID-19 infection and the following: (1) the patients' age, (2) the patients' blood lymphocytic count, (3) the CT-volumetry of the diseased lung parenchyma, (4) the patient's clinical course and prognosis, and finally (5) the final radiological diagnosis.

**Results:**

Multicenter retrospective analyses were conducted between March and June 2021 on 325 adult COVID-19 patients with positive PCR results and negative history of malignant or autoimmune diseases. They included 186 males and 139 females (57.2%:42.8%). Their mean age was 40.42 years ± 14.531 SD. Three consulting radiologists performed CT-grading of the thymus gland (grade 0–3) and CT-severity scoring (CT-SS) of the pathological lung changes in consensus. Two consulting pulmonologists correlated the clinical severity and blood lymphocytic count. Pearson correlation coefficient (*r*) and linear regression analyses were statistically utilized. Sub-involuted thymus (with CT-grade 0:2) was detected in 42/325 patients (12.9%); all of them had a mild clinical course and low CT-SS (0–1). Thymic rebound hyperplasia was the only positive CT-finding in 15/325 patients (4.6%) without pathological lung changes. A weak positive significant correlation was proved between thymic grade and patient's age, clinical course, and CT-SS (*r* = 0.217, 0.163, and 0.352 with *p* ≤ 0.0001, < 0.0001, and 0.002, respectively). A weak negative significant correlation was found between thymic grade and lymphocytic count (*r* = − 0.343 and *p* ≤ 0.0001). A strong positive significant correlation was encountered between clinical severity against patients' age and CT-SS (*r* = 0.616 and 0.803 with *p* ≤ 0.0001).

**Conclusions:**

The presence of sub-involuted thymus or thymic rebound should not be radiologically overlooked in COVID-19 patients. During COVID-19 infection, the presence of sub-involuted thymus with low CT-grading (0–2) was correlated with young age groups, low CT-severity scoring, mild clinical course, and better prognosis (good prognostic factor). It was seldom seen in old hospitalized patients. Atypically, it was also correlated with normal lymphocytic count or even lymphocytosis. The thymic rebound could be the only positive CT-finding even during the absence of lung involvement.

## Background

The thymus gland is a lymphatic organ. It is responsible for the production and maturation of the T cells in children, and hence, it is one of the basic structures of the immune system. It always enlarges in the first 2 decades of life and then involutes in the third decade. It is easily influenced by certain factors, including infections, stress, neoplasm, chemotherapeutic lines, and surgery, with subsequent hypertrophy [1, 2]. Thymic rebound means abnormal partial or complete homogeneous soft tissue enlargement with convex borders and bilobed configuration [3].

One of the theories that may explain lymphopenia that accompanies SARS-COV is the direct attack of the T cells or suppression of the progenitor cells in the bone marrow or thymus gland. Atrophy of the thymus gland could be a sequel of acute infection with depletion of T cells and hence disturbed immune system. This is aggravated in elderly people because of the normal senile involution process [4].

So far, the importance of thymic CT-grading, as well as thymic rebound hyperplasia during COVID-19 infection, was only investigated in a few studies [5]. Conversely, numerous studies discussed the CT features of COVID-19 and pulmonary parenchymal alarming CT signs such as “crazy-paving pattern” [6].

Therefore, this multivariate study aims to evaluate the relation between thymus CT-grading and rebound during COVID-19 infection and the following: (1) the patients' age, (2) the patients' blood lymphocytic count, (3) the CT-volumetry of the diseased lung parenchyma, (4) the patient's clinical course and prognosis, and finally (5) the final radiological diagnosis.

## Methods

### Patients and ethical protocol

Multicenter multivariate retrospective analyses were conducted between March and June 2021 on 325 adult patients proved for COVID-19 infection with positive PCR tests result and negative history for malignant or autoimmune diseases. They included 186 males and 139 females (57.2%:42.8%). Their mean age was 40.42 years ± 14.531 SD.

**Fig. 1:**
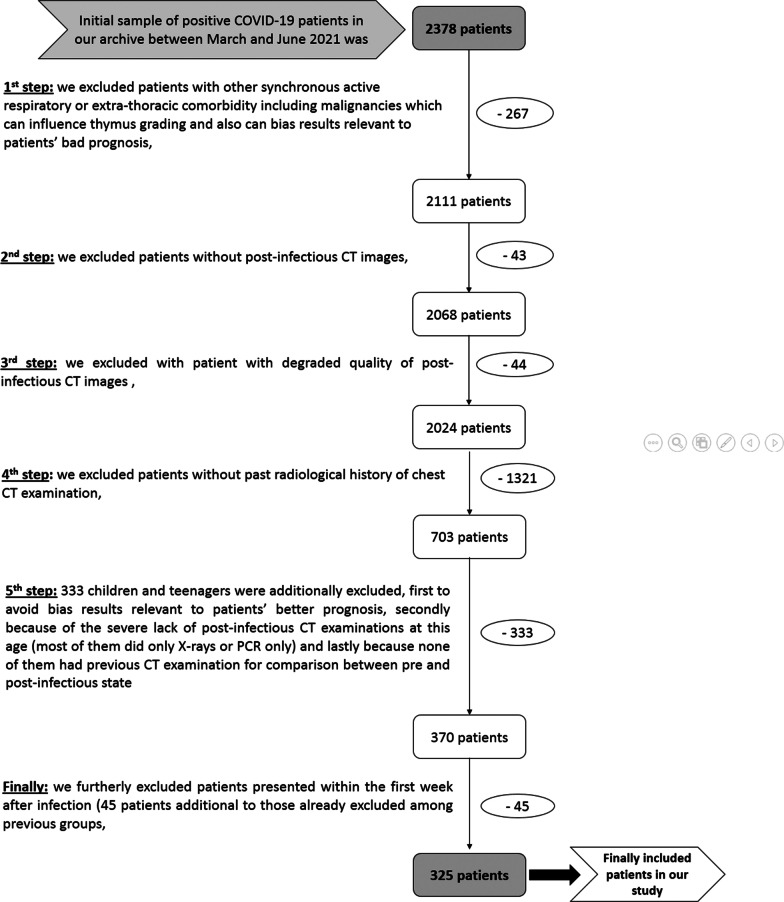
Details of initial sampling, exclusion steps, and final included group in this study

*Details of initial sampling, exclusion steps, and final included group are illustrated in Fig.* 1.

Inclusion criteria were as follows: (1) positive PCR tests confirming COVID-19 infection, (2) acute symptomatic patients during the second week of infection which is considered the critical stage of disease because of the evolution of the cytokine storm and prognosis breaking down, (4) available full medical records including the clinico-laboratory results, (5) available post-infectious multi-detector computed tomography (MDCT) chest examinations to assess thymus CT-grade and CT-severity scoring, and (6) available pre-infectious irrelevant multi-detector computed tomography (MDCT) chest examinations to compare the size and shape of the thymic gland and to determine thymic rebound.

Exclusion criteria were as follows: (1) patients without post-infectious CT images, (2) patients with a degraded quality of post-infectious CT images, (3) patients without previous chest CT examination for comparison, (4) patients with other active respiratory or extra-thoracic comorbidity including malignancies which can influence thymus grading and also can bias results relevant to patients’ bad prognosis, and (5) children and teenagers were additionally excluded, first to avoid bias results relevant to patients' better prognosis, secondly because of the severe lack of post-infectious CT examinations at this age (most of them did only X-rays or PCR only) and lastly because the majority of them did not have any previous CT examination for comparison between pre- and post-infectious state.

The Ethics Committee of the authors’ Faculty of Medicine approved the retrospective study and waived the need for patients' informed consent with the assurance of the confidentiality of patients' data and medical records.

### Medical analysis

Two expert consulting pulmonologists correlated the clinical severity and blood lymphocytic count. They have 19 and 21 years of experience in the field of chest infectious diseases. They applied the WHO criteria for clinical severity of COVID-19 infection based on the clinical symptoms, oxygen saturation, oxygen support, and advanced lines of management [7, 8].

### MDCT scanning (data acquisition and analysis)

The chest CT examination was performed using two MDCT scanners. The first was SOMATOM Sensation 64 (Siemens, Erlangen, Germany). The other was Aquilion CXL/CX 128 (Toshiba, Canon Medical Systems, USA).

The CT-parameters which were applied during the MDCT scanning were as follows: slice thickness (1–1.25 mm), tube rotation (0.6–0.9 s), detector collimation (1 mm), FOV (350 mm × 350 mm), tube voltage (120–130 kVp) according to the body weight, and tube current (200 mA). The intravenous contrast was not administered.

Three consulting radiologists performed the CT-grading of the thymus gland and CT-severity scoring of the affected lung parenchyma and then came to a consensus. They have around 11–16 years of experience in the field of diagnostic MSCT chest imaging.

**Fig. 2:**
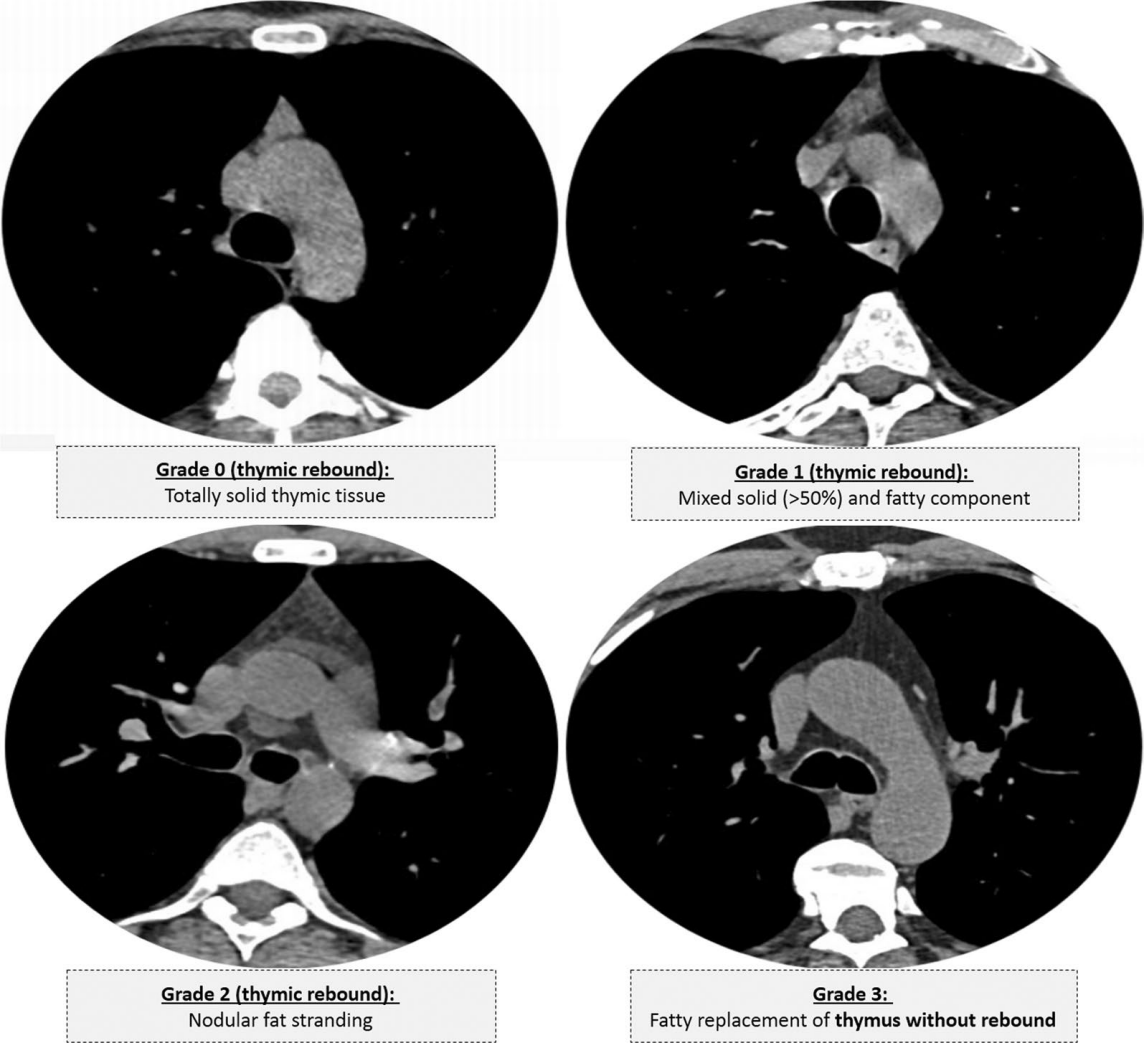
Thymic CT-grading (according to Cuvelier et al. [5]) with provided examples for different CT images with thymic CT-grades (0 to 3) from the current study

They followed Cuvelier et al. [5] in CT-grading of the thymus gland; grade 0 represented isolated solid thymic tissue, grade I represented thymic tissue of mixed predominant solid changes (> 50%) and fat component, and grade 2 represented thymic tissue of mixed predominant fatty component and reticulonodular changes. Grade 3 referred to complete fat replacement and absence of thymic rebound (Fig. 2). Comparison between available pre- and post-infectious chest MDCT examination was performed to evaluate thymic rebound hyperplasia by increased size or change in shape (more lobular pattern).

Chest multi-planar reconstruction (MPR) was performed using the OsiriX MD 11.0 software (Pixmeo SARL, Geneva, Switzerland). ROI 2D/3D reconstruction was used for volumetric lung measurements with threshold-interval adjustment and computed volume calculation. The universal CT-severity scoring was used: Score 1 (0–25%) pathological/normal lung ratio, Score 2 (26–50%), Score 3 (51–75%), and Score 4 (> 75%) [9].

### Statistical analysis

Statistical package for social science (SPSS) version 22 (IBM SPSS Inc., Chicago, IL, USA) was utilized for statistical assessment. Multivariate statistical analyses were performed between six factors (thymus grading, CT-severity scoring, patients' age, sex, clinical grading, and blood lymphocytic levels) including Chi-square analysis (*p* value), Pearson correlation coefficient (*r*), and linear regression analysis.

Other parameters such as the mean, mode, median, variance, and standard deviations were also calculated as needed.

## Results

**Fig. 3:**
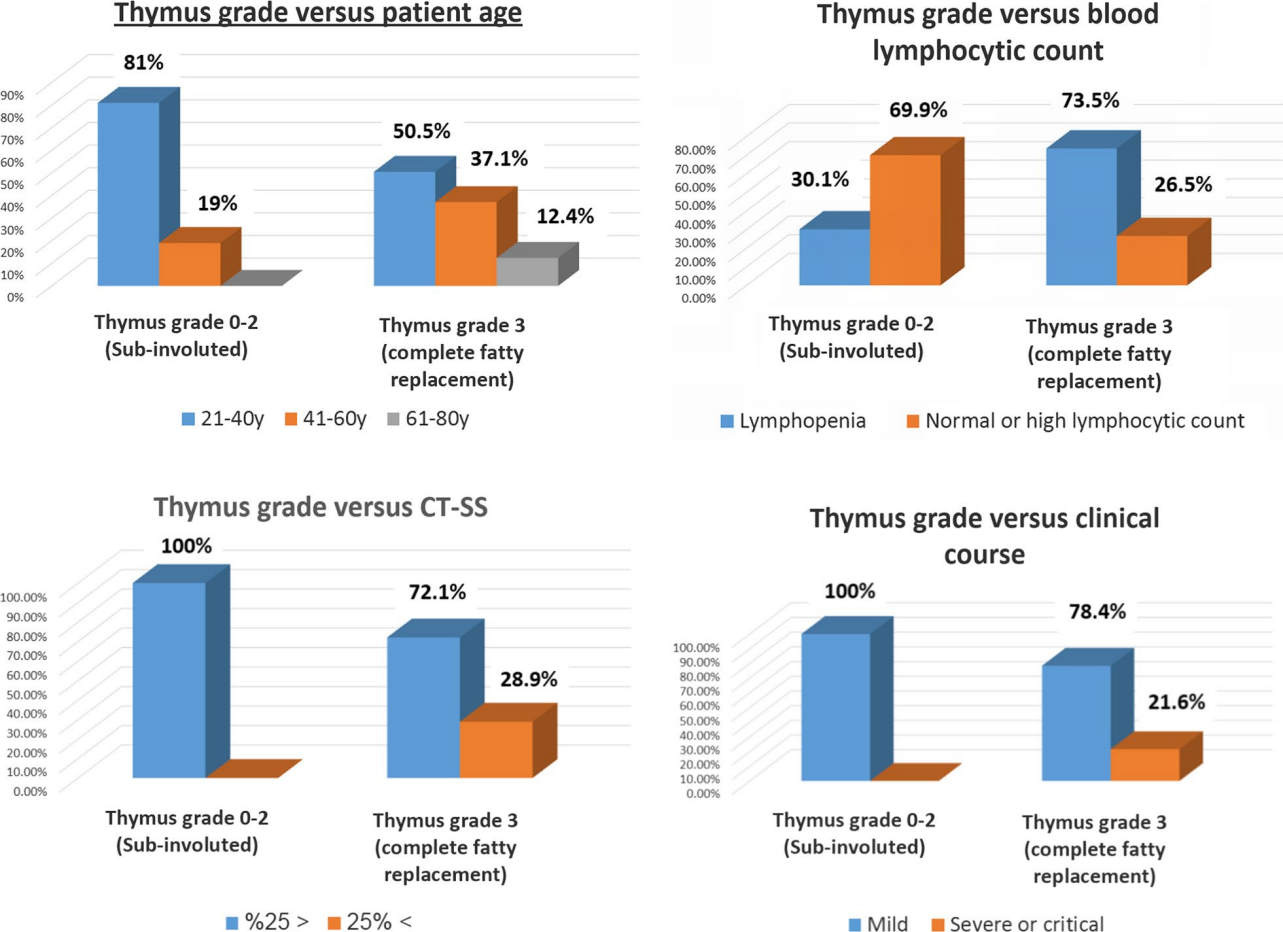
Illustrative isolated charts to show the relation between thymic CT-grading and patients' age, blood lymphocytic count, CT-SS as well as the clinical course

*Illustrative isolated charts show the relation between thymic CT-grading and patients' age, blood lymphocytic count, CT-SS as well as the clinical course* (Fig. 3). An online multivariate chart is additionally demonstrating the distribution frequency of age, sex, thymic CT-grade, lung CT-severity score, clinical course, and blood lymphocytic count among 325 patients included in the study. https://create.piktochart.com/output/55841806-chart-for-detailed-patients-result

### The thymic CT-grading (Table 1)

**Table 1:** Distribution of patients according to thymus CT-grades of involution, patients' age, blood lymphocytic count, CT-severity score, and clinical course during COVID-19 infection

	Sub-involuted thymus (42/325 = 12.9%)			Thymus fatty involution (283/325 = 87.1%)
	Grade 0 (solid)	Grade 1 [solid (> 50%) and fat)	Grade 2 [solid and fat (> 50%)] = Reticulonodular pattern)	Grade 3 (complete fatty replacement)
*Total number*	6/325	7/325	29/325	283/325
*Age*				
20–40 years	6 (100%) (*P* = 0.024)**	7 (100%) (*P* = 0.014)**	21 (72.4%) (*P* = 0.042)**	143 (50.5%)
41–60 years	0 (*P* = 0.071)	0 (*P* = 0.055)	8 (27.6) (*P* = 0.395)	105 (37.1%)
61–80 years	0 (*P* = 0.390)	0 (*P* = 0.353)	0 (*P* = 0.05)	35 (12.4%)
*Lymphocytes*				
Lymphopenia	0 (*P* = 0.0001)**	0 (*P* = 0.00004)**	13 (44.8%) (*P* = 0.005)**	208 (73.5%)
Normal or lymphocytosis	6 (100%) (*P* = 0.0001)**	7 (100%) (*P* = 0.00004)**	16 (45.2%) (*P* = 0.005)**	75 (26.5%)
*Lung CT-score*				
0% (Clear lung)	4 (66.7%) (*P* ≤ 0.0001)**	4 (57.1%) (*P* ≤ 0.0001)**	7 (24.1%) (*P* ≤ 0.0001)**	0
1–25%	2 (33.3) (*P* = 0.04)	3 (42.9%) (*P* = 0.096)	22 (75.9%) (*P* = 0.551)	204 (72.1%)
25–50%	0 (*P* = 0.259)	0 (*P* = 0.222)	0 (*P* = 0.01)**	56 (19.8%)
51–75%	0 (*P* = 0.574)	0 (*P* = 0.505)	0 (*P* = 0.157)	19 (6.7%)
76–100%	0 (*P* = 0.783)	0 (*P* = 0.765)	0 (*P* = 0.529)	4 (1.4%)
*Symptoms*				
Mild	6 (100%) (*P* = 0.235)	7 (100%) (*P* = 0.199)	29 (100%) (*P* = 0.007)**	222 (78.4%)
Severe	0 (*P* = 0.235)	0 (*P* = 0.199)	0 (*P* = 0.007)**	61 (21.6%)

**Correlation is significant at the 0.05 level

The sub-involuted thymus (CT-grade 0–2) was depicted in 42/325 (12.9%) of patients. Among them, 6/42 (14.3%) expressed grade 0, while 7/42 (16.7%) expressed grade 1 and 29/42 (69.1%) expressed grade 2.

On the other hand, 283/325 (87.1%) of patients showed total involution with complete fat replacement and absence of thymic rebound (grade 3).

### The demographic data and clinical severity (Table 1)

The age of 54.5% of patients in this study ranged between 20 and 40 years, while the age of 34.8% of patients ranged between 40 and 60 years and only 28% of patients were above 60 years.

While 72.3% of patients in this study were clinically mild regarding the grade of dyspnea, oxygen saturation in room air, and oxygen therapeutic requirements, on the other hand 27.7% of them were clinically severe and hospitalized.

All patients with sub-involuted thymus (CT-grade 0–2) were clinically mild; meanwhile, all severe or critical hospitalized patients showed grade 3 thymus (complete fat replacement without rebound).

### The lymphocytic levels (Table 1)

221/325 (68%) of patients in this study had low blood lymphocytic count (lymphopenia). On the other hand, only 104/325 (32%) of patients had normal or elevated lymphocytic count (lymphocytosis).

Strikingly, 29/42 (69%) of patients with sub-involuted thymus (grade 0–2) showed normal or elevated lymphocytic count (lymphocytosis).

### The CT volumetric scoring (Table 1)

231/325 (71.1%) of patients scored 1 with mild clinical course. On the other hand, 56/325 (17.2%) of patients scored 2, while 19/325 (5.8%) of patients scored 3, and 4/325 (1.2%) of patients scored 4. This corresponded to severe or critical clinical course.

**Fig. 4:**
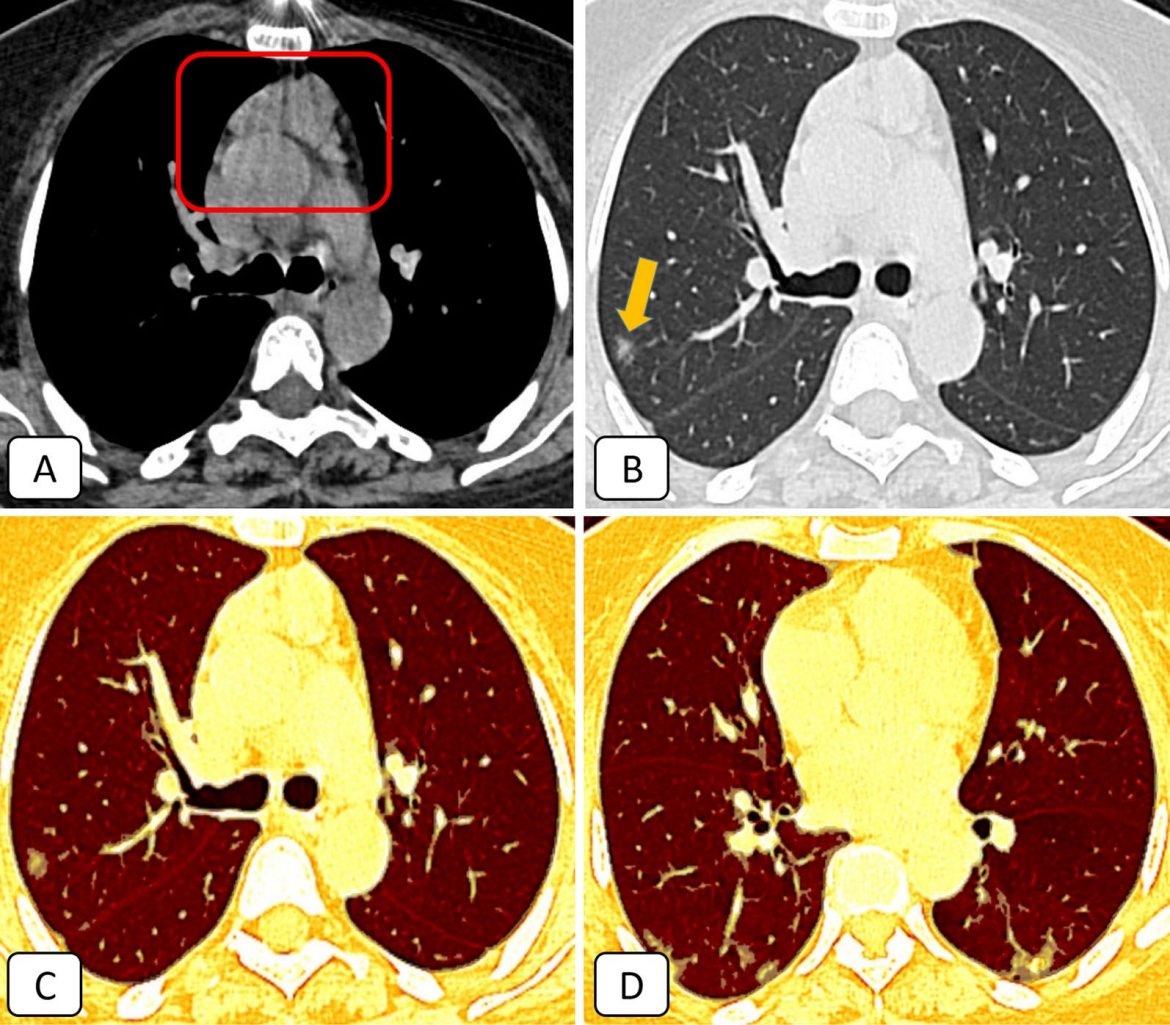
A 57-year-old female COVID-19 patient complained of loss of taste and smell: **A** Axial mediastinal window CT showing solid soft tissue replacement of the thymic gland (grade 0) [red square]. **B** Axial lung window CT showed sub-pleural small ground-glass nodular opacity in the posterior segment of the right upper lobe (orange arrow). **C**–**D** Axial 3D volumetry cuts highlighted the bilateral basal small sub-pleural ground-glass opacities. The pathological lung volume was around 1% of the total lung volume

**Fig. 5:**
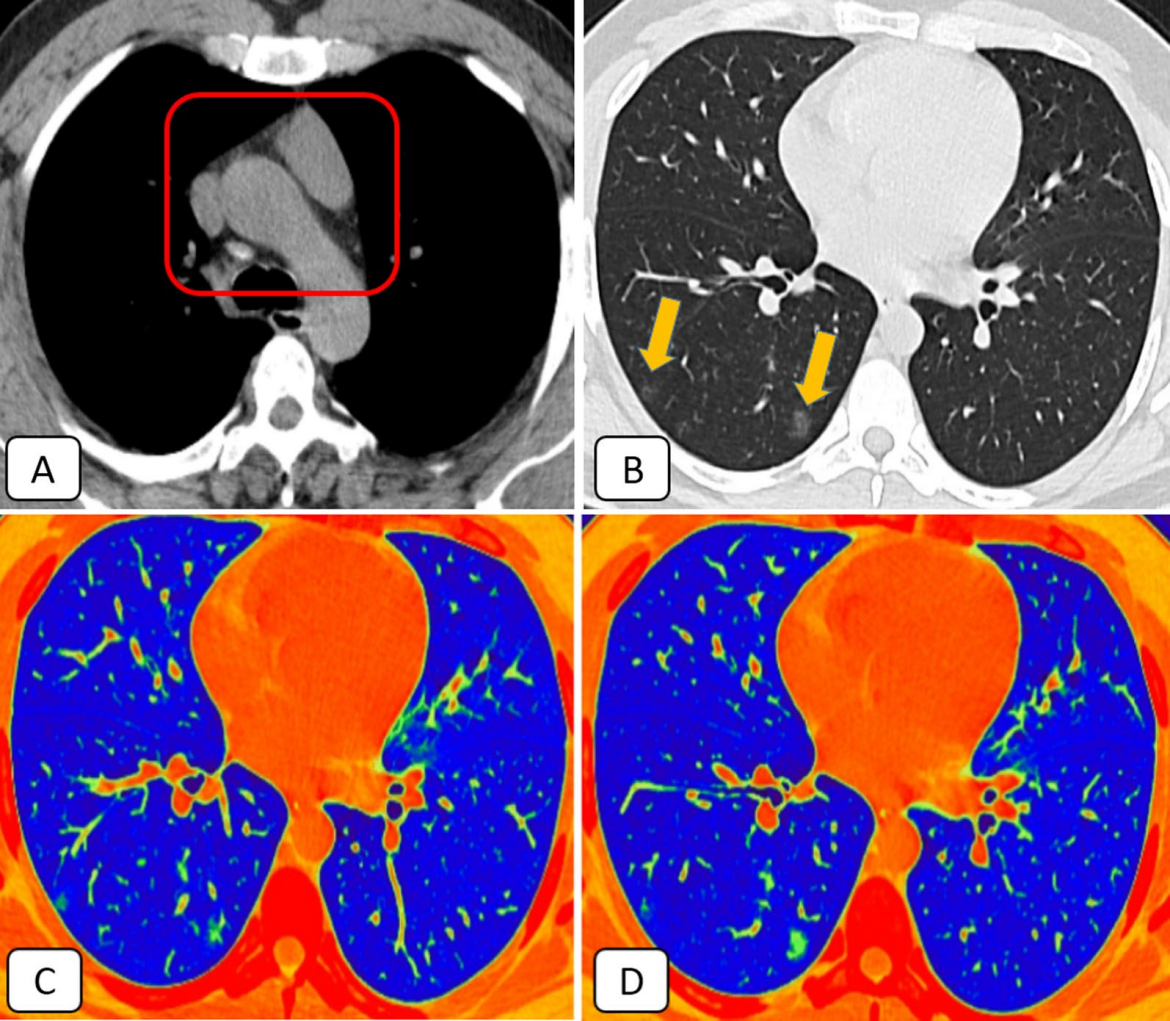
A 53-year-old male COVID-19 patient complained of fever, cough, loss of taste and smell: **A** Axial mediastinal window CT showed mixed soft tissue and fatty replacement of the thymic gland. The solid component is > 50% (grade 1) [red square]. **B** Axial lung window CT showed two right basal sub-pleural small ground-glass nodular opacities (orange arrows). **C**–**D** Axial 3D volumetry cuts highlighted the right basal small sub-pleural ground-glass opacities. The pathological lung volume was around 0.5% of the total lung volume

**Fig. 6:**
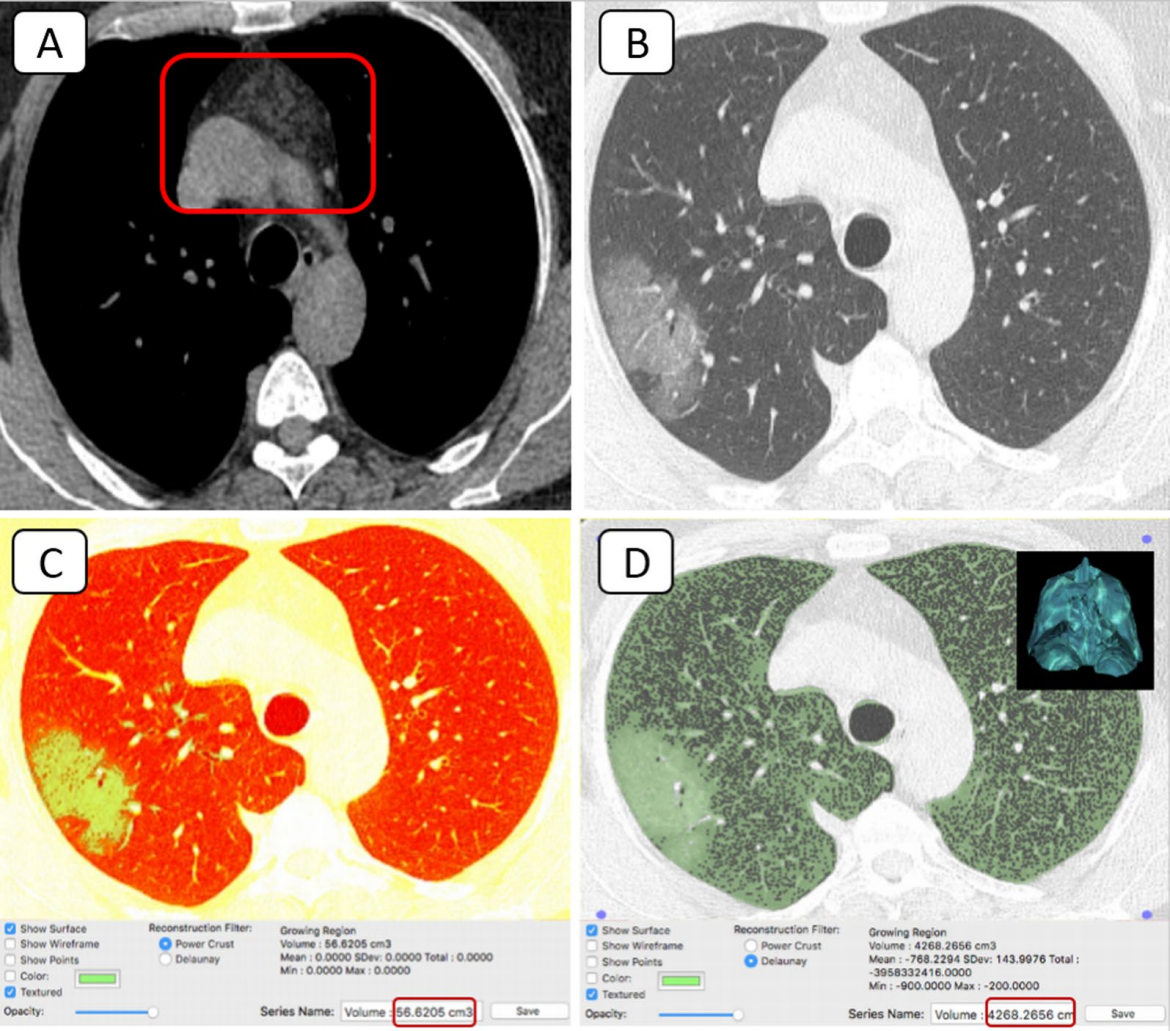
A 48-year-old male COVID-19 patient complained of fever, dry cough, and chest pain: **A** Axial mediastinal window CT showed nodular infiltration of the thymic gland (grade 2) [red square]. **B** Axial lung window CT showed a right upper lobar sub-pleural ground-glass patch. **C**–**D** Axial 3D volumetry cuts highlighted the right sub-pleural ground-glass patch. The pathological lung volume was around 1.3% of the total lung volume

27/42 (64.3%) of patients with sub-involuted thymus (CT-grade 0–2) scored 1 with mild clinical course (Figs. 4, 5, 6).

#### Thymic rebound

**Fig. 7:**
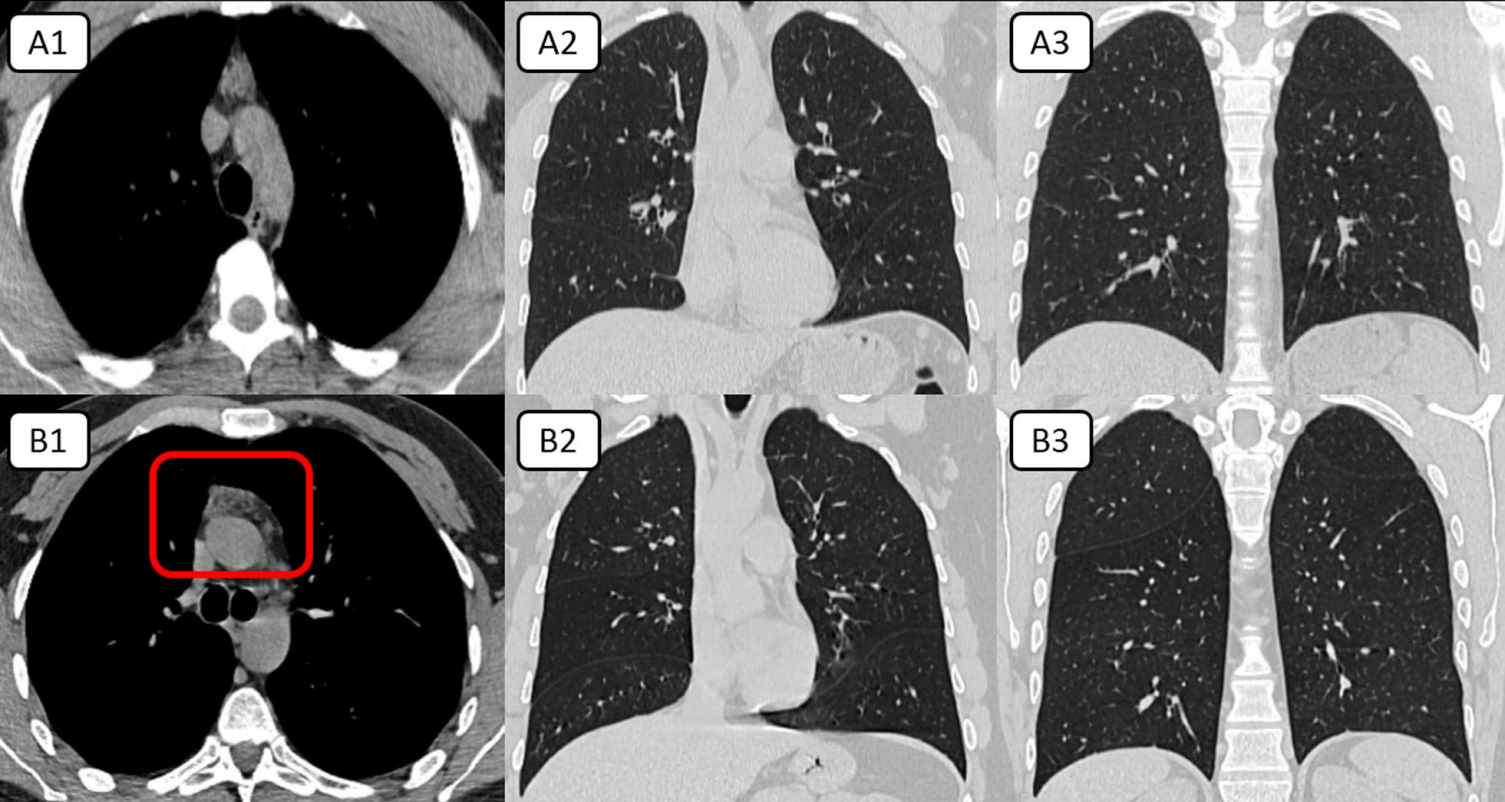
**Two COVID-19 patients proved with PCR test results without lung involvement:** (**A**) 31-year-old male patient complained from fever and cough for two days; [A1]: Axial mediastinal window CT showing solid soft tissue replacement of the thymic gland (grade 0). [A2-3]: Axial lung window CT cuts at the level of the cardiac silhouette and para-vertebral planes show clear bilateral lung parenchyma. (**B**) A 33-year-old male patient complained of fever, cough, and chest pain for three days; [B1]: Axial mediastinal window CT showing a nodular pattern of the thymic gland (grade 2). [B2-3]: Axial lung window CT cuts at the level of the cardiac silhouette and para-vertebral planes show clear bilateral lung parenchyma

15/325 (4.6%) of patients in this study strikingly showed clear lung parenchyma. All of them showed sub-involuted thymus (CT-grade 0–2) and CT signs of thymic rebound hyperplasia manifested by progression in size and more lobular shape after comparison between the pre- and post-infectious CT examinations (Fig. 7).

#### The multivariate Chi-square analysis (*p* value), Pearson correlation coefficient (*r*), and linear regression analyses (Table 2)

**Table 2:** Multivariate SPSS analysis including Pearson correlation and linear regression significant analyses

	Thymus	Symptoms	CTSI	Lymphocytes	Age	Sex
*Thymus*						
Pearson Correlation	1	0.163**	0.352**	− 0.343*	0.217**	0.078
Sig. (1-tailed)		0.002^∞^	0.000^∞^	0.000^∞^	0.000^∞^	0.081
N	325	325	325	325	325	325
*Symptoms*						
Pearson Correlation	0.163**	1	0.803***	− 0.023**	0.616***	0.049
Sig. (1-tailed)	0.002^∞^		0.000^∞^	0.343	0.000^∞^	0.188
N	325	325	325	325	325	325
*CTSI*						
Pearson Correlation	0.352**	0.803***	1	− 0.074	0.533***	0.035
Sig. (1-tailed)	0.000^∞^	0.000^∞^		0.090	0.000^∞^	0.265
N	325	325	325	325	325	325
*Lymphocytes*						
Pearson Correlation	− 0.343*	− 0.023	− 0.074	1	− 0.026	− 0.039
Sig. (1-tailed)	0.000^∞^	0.343	0.090		0.321	0.240
N	325	325	325	325	325	325
*Age*						
Pearson Correlation	0.217**	0.616***	0.533***	− 0.026	1	0.006
Sig. (1-tailed)	0.000^∞^	0.000^∞^	0.000^∞^	0.321		0.455
N	325	325	325	325	325	325
*Sex*						
Pearson Correlation	0.078	0.049	0.035	− 0.039	0.006	1
Sig. (1-tailed)	0.081	0.188	0.265	0.240	0.455	
N	325	325	325	325	325	325

No correlation (*r* =  − 0.1:0.1)

*Weak negative correlation (*r* =  − 0.1: − 0.5). **Weak positive correlation (*r* = 0.1:0.5). ***Strong positive correlation (*r* > 0.5)

^∞^Correlation is significant at the *P* = 0.01 level (1-tailed)

**Fig. 8:**
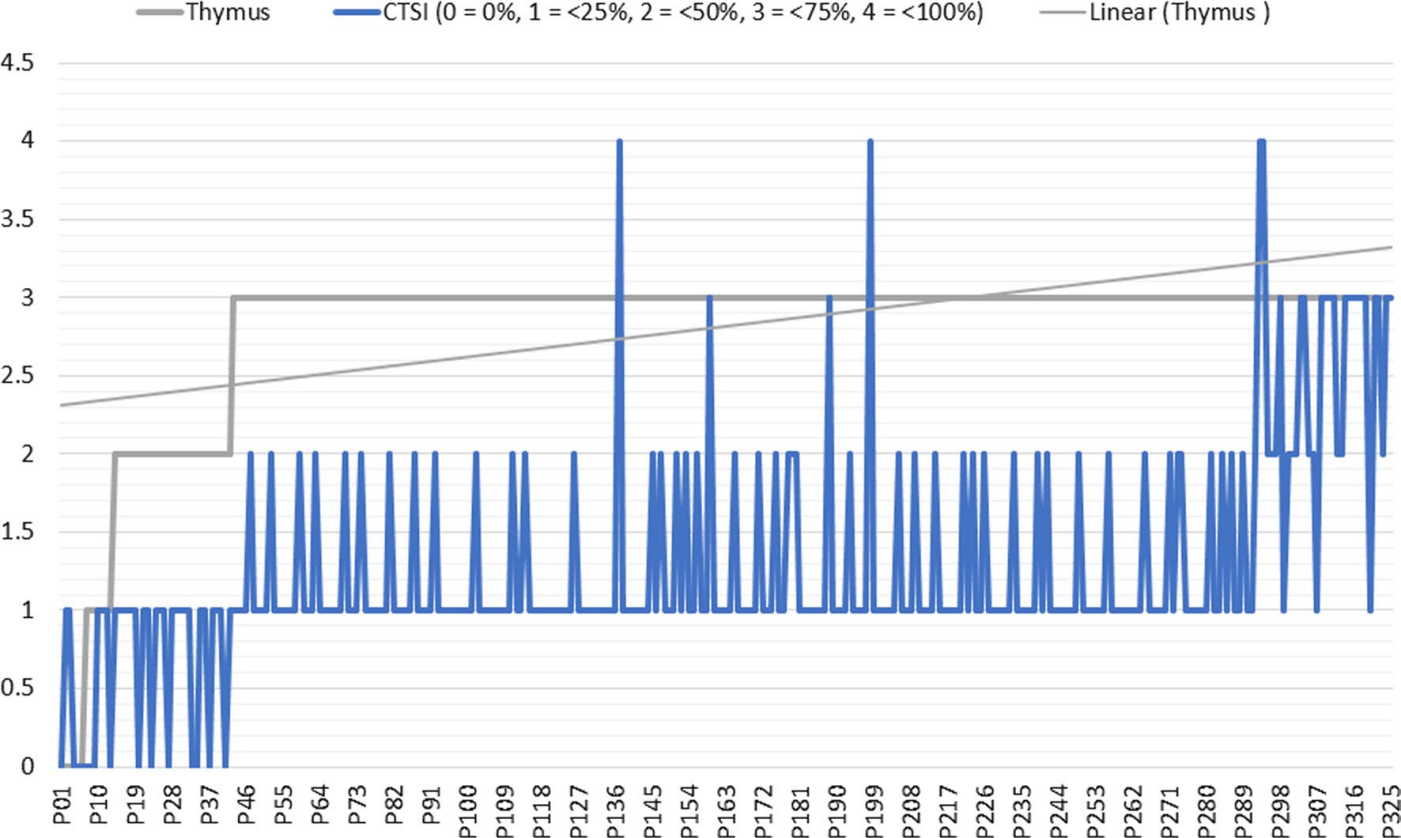
Bivariate chart demonstrating the distribution frequency and linear relation between thymic CT-score and lung CT-severity score: High CT-severity scores occur more with grade III thymic CT-score

**Fig. 9:**
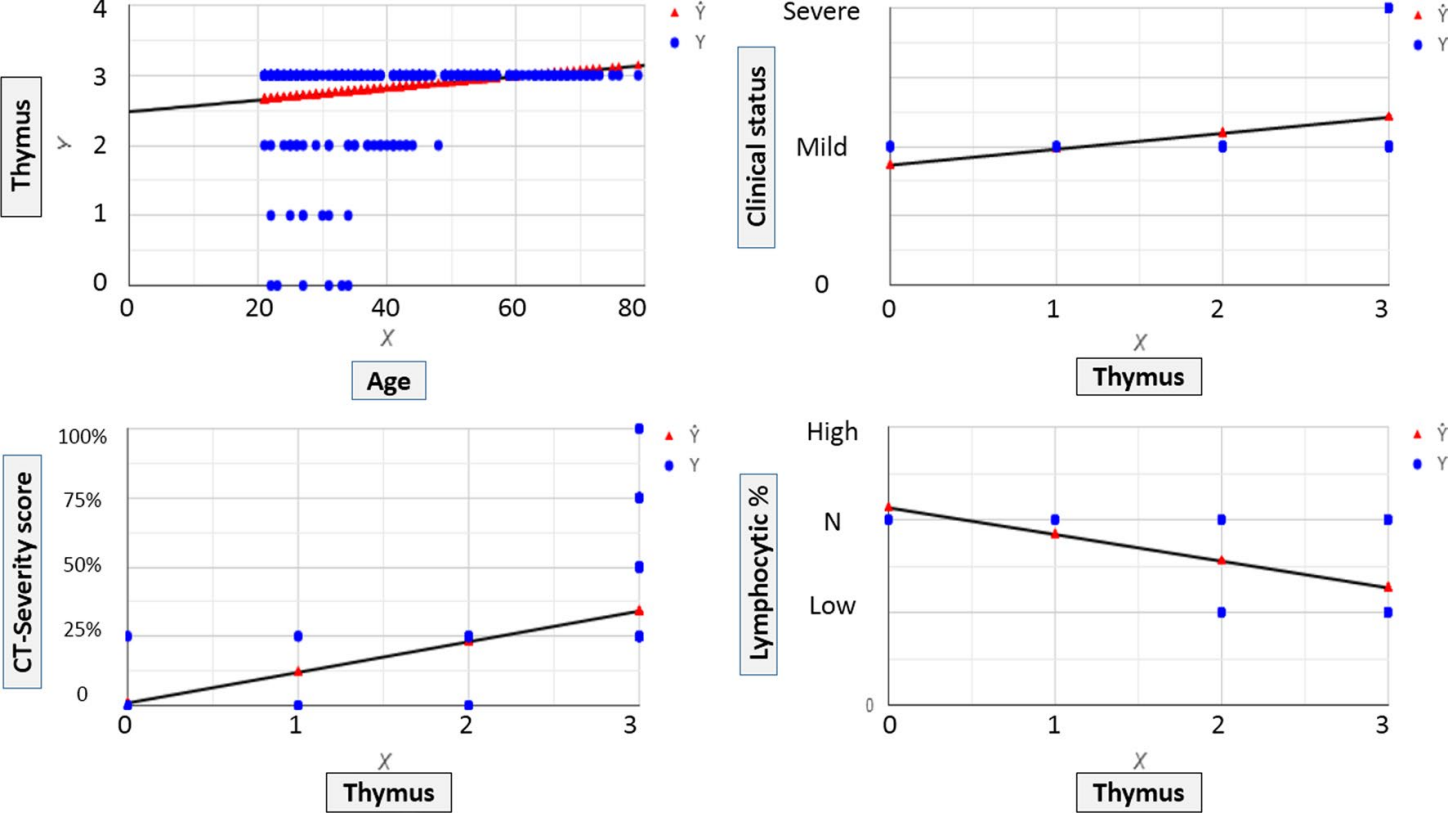
Multivariate chart demonstrating the linear regression curves between the thymic CT-score and age, clinical course, lung CT-severity score, and blood lymphocytic count. Only the curve between the thymic score and blood lymphocytic count showed their reversed relation

A weak positive significant correlation was encountered between thymic grade and patient's age, clinical course, and CT-severity score (*r* = 0.217, 0.163, and 0.352 with *p* ≤ 0.0001, < 0.0001, and 0.002 , respectively) (Figs. 8 and 9).

A weak negative significant correlation was found between thymic grade and lymphocytic count (*r* = − 0.343 and *p* ≤ 0.0001) (Figs. 8 and 9).

A strong positive significant correlation was proved between clinical severity against patients' age and CT-severity scoring (*r* = 0.616 and 0.803 with *p* ≤ 0.0001) (Figs. 8 and 9).

**Fig. 10:**
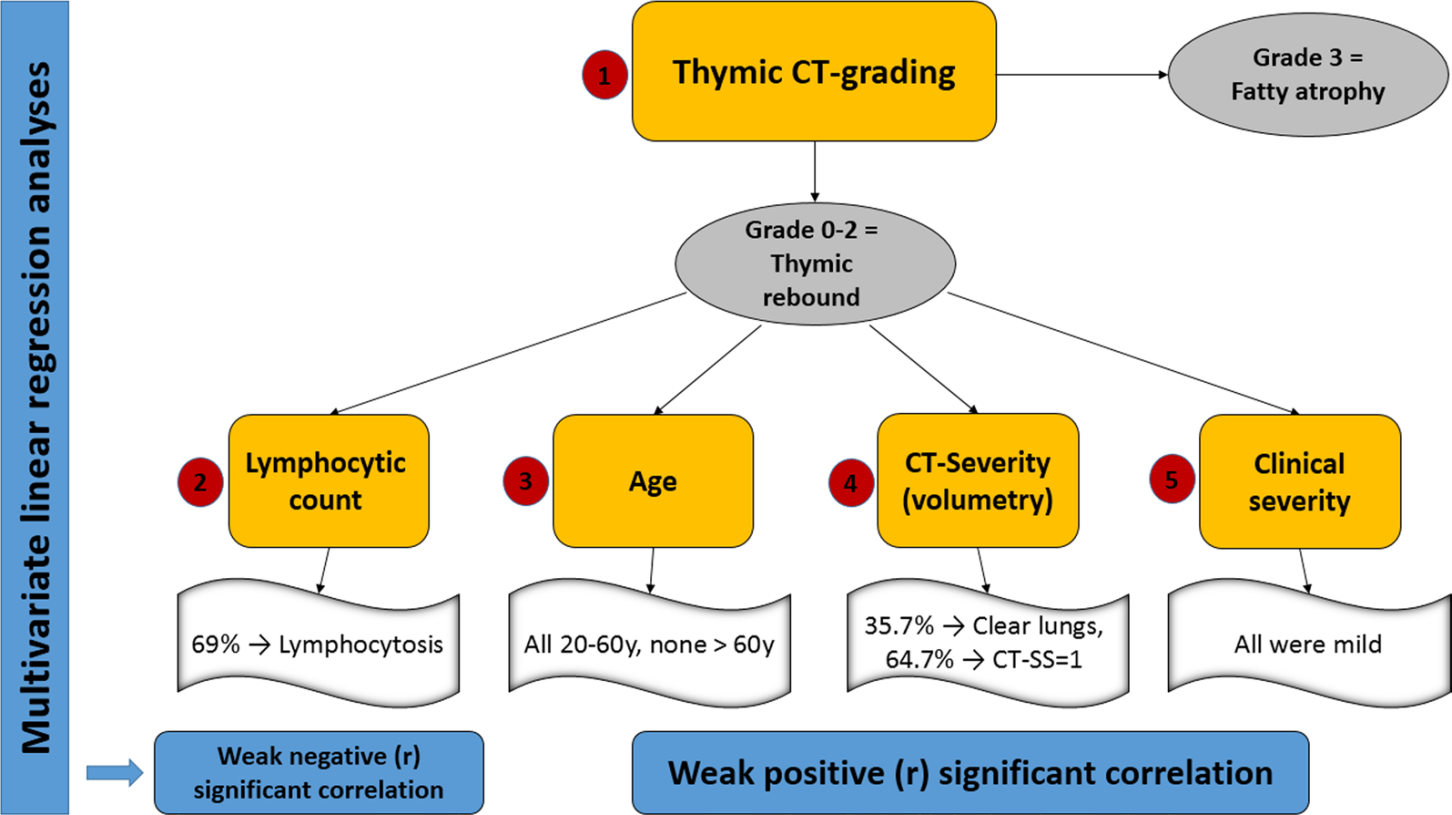
A graph is summarizing the results of this multivariate linear regression analyses of

A graph is summarizing the results of the multivariate linear regression analyses of (1) thymic CT-grading, (2) blood lymphocytic count, (3) age of patients, (4) CT-severity scoring (volumetry), and (5) clinical severity (Fig. 10).

## Discussion

The role of chest CT in the evaluation of COVID-19 patients had expanded a lot since the announcement of the pandemic, particularly in those with severe or critical clinical symptoms and signs. However, only a few types of research were directed toward the impact of thymic gland enlargement on the clinical severity and prognosis of the disease as well as the associated laboratory markers such as the lymphocytic count [5].

In this study, multivariate linear regression analysis was performed to answer several questions about this relation. This analysis statistically proved a protective function for the thymus gland during COVID-19 infection.

This agreed with Cuvelier et al. [5] who concluded that the presence of thymic enlargement in COVID-19 patients could be considered a protective factor that adapts to the lymphocytic depletion yielding a better prognosis; meanwhile, the absence of this thymic enlargement or reactivity especially in older patients could be an additional explanation to the bad prognosis beside the presence of comorbidities and COVID-19-related vasculopathy. This also goes side by side with the speculation of Güneş H et al. [10] regarding the protective role of the thymus gland in combating COVID-19 infection in children giving hope for a possible additional treatment that can stimulate or prevent the inhibition of the thymus gland. This principle was also discussed by Kellogg et al. [11] who additionally provided the impact of this relation on antibody treatments and immunization. He concluded that COVID-19 patients with poor thymic function are advised to be prophylactically treated with recombinant antibodies or convalescent serum; meanwhile, they need higher doses of COVID-19 vaccinations.

In this study, the sub-involuted thymus (with CT-grade 0–2) was predominantly noticed among COVID-19 patients between 20 and 40 years and to a lesser extent between 40 and 60 years. They showed normal or increased lymphocytic count, mild clinical course, low CT-severity score, and a better prognosis. On the other hand, patients above 60 years old showed total thymus involution with complete fat replacement. Particularly this group of old patients showed high CT-severity score (CT-SS) with severe clinical symptoms and signs.

This is typically matching the results and conclusion of Chen et al. [12] who reported that 64.7% of their included patients had complete fat replacement and correlated this thymic involution to poor prognosis in COVID-19 patients above 40 years (OR = 3.071, *P* = 0.000).

Lymphopenia is one of the typical laboratory landmarks for COVID-19 infection. Normal lymphocytic count or lymphocytosis was seldom discovered among COVID-19 patients [6].

In this study, lymphopenia was predominant with poor prognosis. Meanwhile, the majority of the other patients who had normal lymphocytic count or even lymphocytosis showed sub-involuted thymus (CT-grade 0–2) with or without thymic rebound enlargement and expressed better clinical outcomes.

This inversed relation between lymphocytic count and thymic CT-scoring was also statistically confirmed by Cuvelier et al. [5] (*r* = 0.56, *p* = 0.007). Also, the inversed relation between lymphocytic count and patients' poor prognosis was statistically confirmed by Cakmak et al. [13] (*p* = 0.001).

This study statistically proved a strong triad positive correlation between the clinical severity, CT-severity score, and old age.

This fact was already established by several studies such as Leonardi et al. [14], Yang et al. [15], and Li et al. [16] using different CT scoring systems and ROC analysis. Leonardi et al. [14] statically correlated a CT-severity score (23%) with critical disease (*r* = 0.982). Yang et al. [15] correlated a CT-severity score (19.5/40) with severe disease (*r* = 0.892). Li et al. [16] correlated a CT-severity score (7.5/20) with severe/critical disease (*r* = 0.918).

In this study, thymic rebound hyperplasia was found among few COVID-19 patients without any pathological lung changes.

This is in keeping with Sabri et al. [17] who found thymic enlargement in 14.3% of their patients. Therefore, the presence of thymic rebound should never be overlooked by any radiologist.

## Strength and limitations

Using a novel multivariate analysis, this study adds to the previous literature which described the impact of thymic enlargement on the clinical course and prognosis of COVID-19 infection. Additionally, it correlated the thymus CT-grade with other factors such as patient age and blood lymphocytic count at the same time.

However, the non-availability of most of the patients for long-term follow-up for the post-COVID disease was a limitation in this study. We recommend further long-term future studies on this issue.

## Conclusions

The presence of sub-involuted thymus or thymic rebound should not be radiologically overlooked in COVID-19 patients. During COVID-19 infection, the presence of sub-involuted thymus with low CT-grading (0–2) was correlated with young age groups, low CT-severity scoring, mild clinical course, and better prognosis (good prognostic factor). It was seldom seen in old hospitalized patients. Atypically, it was also correlated with normal lymphocytic count or even lymphocytosis. The thymic rebound could be the only positive CT-finding even during the absence of lung involvement.

## Data Availability

The datasets used and/or analyzed during the current study are available from the corresponding author on reasonable request.

## Abbreviations

COVID-19: Novel coronavirus disease (2019)
MDCT: Multi-detector computed tomography
PCR: Polymerase chain reaction.
CT-SS: Computed tomography-severity scoring
